# Frequency of Painful Crisis and Other Associated Complications of Sickle Cell Anemia Among Children

**DOI:** 10.7759/cureus.48115

**Published:** 2023-11-01

**Authors:** Jana S Sendy, Mohammed S Alsadun, Sarah S Alamer, Shujon M Alazzam, Mansour M Alqurashi, Adel H Almudaibigh

**Affiliations:** 1 College of Medicine, Almaarefa University, Riyadh, SAU; 2 Pediatric Cardiology, Almaarefa University, Riyadh, SAU; 3 Pediatric Hematology, King Saud Medical City, Riyadh, SAU

**Keywords:** sickle cell disease (scd), hydroxyurea, vaso-occlusive crisis, sickle cell anemia, pediatric

## Abstract

Background

Sickle cell disease (SCD) represents a group of inherited health conditions that affect red blood cells. SCD is a relatively common genetic disorder in Saudi Arabia, with the highest prevalence found in the Eastern Province region. The most common complications of SCD include acute chest syndrome, vaso-occlusive crisis, stroke, and avascular necrosis of the femoral head. The disease itself is not a cause of mortality but systemic complications are.

Methodology

In this retrospective study, we aimed to determine the frequency of painful crisis and the associated complications of sickle cell anemia (SCA) among children at King Saud Medical City (KSMC) in Riyadh, Saudi Arabia.

Results

This study included a total of 70 children with SCA below the age of 14 years who were admitted to KSMC from January 2021 to December 2021. Overall, 60% of the participants had one painful crisis attack per year, whereas 27% had two attacks. Furthermore, 94% of the participants were being treated with hydroxyurea. The most frequent cause of admission was painful crises with acute chest syndrome.

Conclusions

This study highlights the frequency of hydroxyurea use among SCA patients. Our results showed that participants who developed one to two painful crises per year were hospitalized for four to nine days on average with increased utilization of hydroxyurea.

## Introduction

Acute painful crises are recurrent episodes of severe pain due to vaso-occlusion of small blood vessels. Sickle cell disease (SCD) describes a single mutation of inherited health conditions that affect red blood cells (RBCs). SCD is caused by homozygous mutations at the sixth codon of the* β-globin* gene in the β-globin cluster (HBB) on chromosome 11 [1]. The most serious type of SCD is called sickle cell anemia (SCA) [2]. In SCA, RBCs are abnormally sickle-shaped [3] and are known as sickle cells. Sickle cells can block small blood capillaries causing vaso-occlusive crisis (VOC) and ischemia in tissues and organs [4]. SCD affects approximately 72,000 people in the United States, with 2 million being carriers of the disease [5]. In Africa, more than 200,000 infants are born with SCD every year [6]. In Saudi Arabia, data on the prevalence of SCD shows marked regional variation, with the highest prevalence in the Eastern (134.1 per 1000) and the lowest in the Northern (13.5 per 1,000) provinces [7]. In Al Madinah, the Western province, the estimated prevalence of sickle cell homozygosity (Hb SS) is 0.01%, and that of the carrier state (Hb AS) is 0.09% [8]. SCA affects nearly all organs and has many complications, such as painful episodes of VOC, acute chest syndrome (ACS), and stroke [9]. Complications related to SCA are associated with high rates of morbidity and mortality [10]. A recent study conducted in Makkah found that VOC was the second most prevalent complication among pediatric patients with SCA [11]. Advances in medical and supportive care have resulted in changes in the cause of hospitalization among children with SCD over time [12]. In one study, hospitalization was more prolonged among children with infection and ACS, where medication non-compliance was the main underlying factor for hospital admission and prolongation [13]. A study aimed to determine the incidence of VOC and the role of treatment. It showed that puberty was delayed in children who suffered from SCA, whereas there were some improvements in patients receiving hydroxyurea (HU) in the Saudi pediatric population [14]. There has been an improvement in the use of HU for the care and treatment of children with SCD. The safety and efficacy of HU have been well documented in the literature regarding its use in children and adults, as well as in reducing acute complications, such as VOC, ACS, blood transfusions, and hospitalizations [14] SCD is a potentially life-threatening disorder that is associated with many complications interfering with daily activities and the quality of life of patients.

In this study, we aimed to determine the frequency of painful crises and the associated complications of SCA among children at King Saud Medical City (KSMC) in Riyadh, Saudi Arabia. In addition, we aimed to assess other complications of SCA during painful crises and evaluate the use of HU among children at KSMC. Because of the high frequency of children with SCA visiting the hematology clinic, further investigations into their painful crises will help detect such severe complications and improve their quality of life. We expected that children with SCA attending the hematology clinic and suffering from complications of painful crisis would develop other serious health complications.

## Materials and methods

Study design, setting, and participants

This retrospective study was conducted at the pediatric department of KSMC, Riyadh, Saudi Arabia. The inclusion criteria were children with SCA below the age of 14 years who were admitted to KSMC hospitals from January 2021 to December 2021. We excluded patients with incomplete data and patients with sickle cell trait. This study was approved by the Research Ethics Committee of KSMC in Riyadh and Almaarefa University College of Medicine (approval numbers: KACST,KSA:H-01-R-053 and HA-01-R-064). We obtained patients’ data from the medical files.

Methodology

We obtained clinical data by reviewing patients’ hospital electronic files. Data were collected and entered into a Microsoft Excel (Microsoft Corp., Redmond, WA, USA) spreadsheet, including sociodemographic data (age, sex, nationality), duration of hospitalization, number of admissions due to painful crises, number of painful crisis attacks, associated complications, chronic diseases, and medication use (HU). We analyzed the data using SPSS (IBM Corp., Armonk, NY, USA). A p-value of less than 0.05 was considered statistically significant.

## Results

This retrospective study included 70 pediatric patients with SCA who presented with a painful crisis. Table 1 shows that the most common age group was 10-14 years (34%), followed by 8-10 years (29%). Overall, 53% were female and 47.1% were male. Of the total study population, 93% were Saudi.

**Table 1:** Sociodemographic data of the study participants.

Variables	Frequency	Percentage
Age (years)		
Less than 5	8	11.4%
5–7	18	25.7%
8–10	20	28.6%
10–14	24	34.3%
Gender		
Male	33	47.1%
Female	37	52.9%
Nationality		
Saudi	65	92.9%
Non-Saudi	5	7.1%

Table 2 shows that 60% of the patients had one attack per year and 27% had two attacks per year.

**Table 2:** Number of attacks of painful crises per year among the study participants.

Number of attacks of painful crises per year	Frequency	Percentage
One attack	42	60%
Two attacks	19	27.1%
Three attacks	5	7.2%
Four attacks	4	5.7%
Total	70	100%

As shown in Table 3, 31% of the patients were hospitalized for less than four days due to one painful crisis attack per year, while 45% were hospitalized for four to nine days. On the other hand, 59% of the patients who had experienced two attacks of painful crises were hospitalized for four to nine days for the first attack and 53% were hospitalized for four to nine days for the second attack.

**Table 3:** Duration of hospitalization among the study participants with only one painful crisis attack per year.

Duration of hospitalization (days)	Frequency	Percentage
Less than 4	13	31%
4–9	19	45.2%
10–15	9	21.4%
More than15	1	2.4%
Total	42	100%

**Table 4:** Duration of hospitalization among the study participants with two painful crisis attacks per year.

Attack number	Duration of hospitalization (days)				Total
	Less than 4	4–9	10–15	More than15	
One	2 (10.5%)	11 (57.9%)	5 (26.3%)	1 (5.3%)	19 (100%)
Two	8 (42.1%)	10 (52.6%)	1 (5.3%)	0 (0%)	19 (100%)

Table 5 shows the duration of hospitalization due to three attacks of painful crises per year. Overall, 60% of patients during the first attack were hospitalized for four to nine days while 40% of the patients during the second attack were hospitalized for less than four days. A similar percentage of patients were hospitalized for four to nine days, and 100% of the patients were hospitalized for four to nine days during the third attack.

**Table 5:** Duration of hospitalization among the study participants with three painful crisis attacks per year.

Attack number	Duration of hospitalization (days)				Total
	Less than 4	4–9	10–15	More than15	
One	1 (20%)	3 (60%)	1 (20%)	0 (0%)	5 (100%)
Two	2 (40%)	2 (40%)	1 (20%)	0 (0%)	5 (100%)
Three	0 (0%)	5 (100%)	0 (0%)	0 (0%)	5 (100%)

Table 6 shows the duration of hospitalization due to four attacks of painful crises. During the first attack, 75% were hospitalized for four to nine days. During the second and third attacks, 50% were hospitalized for four to nine days. During the fourth attack, 100% were hospitalized for four to nine days.

**Table 6:** Duration of hospitalization among the study participants with four painful crisis attacks per year.

Attack number	Duration of hospitalization (days)				Total
	Less than 4	4–9	10–15	More than15	
One	0 (0%)	3 (75%)	1 (25%)	0 (0%)	4 (100%)
Two	2 (50%)	2 (50%)	0 (0%)	0 (0%)	4 (100%)
Three	2 (50%)	2 (50%)	0 (0%)	0 (0%)	4 (100%)
Four	0 (0%)	4 (100%)	0 (0%)	0 (0%)	4 (100%)

Table 7 explains the associated complications during painful crises for children with SCA. The highest percentage of 31% was noted for ACS, 23% for osteomyelitis, 19% for stroke, and 12% for splenic sequestration. As shown in Table 8, the use of HU during painful crises was detected among 94%, while 6% were not administered HU.

**Table 7:** Complications associated with painful crises among study participants. ACS = acute chest syndrome; AVN = avascular necrosis; OM = osteomyelitis

Complication	Frequency	Percentage
ACS	8	30.7%
ACS + SQ	1	3.9%
AVN	1	3.9%
OM	6	23%
OM + ACS	1	3.9%
Priapism	1	3.9%
SQ	3	11.5%
Stroke	5	19.2%
Total	26	100%

**Table 8:** Use of hydroxyurea as medication during painful crises among study participants.

Hydroxyurea use	Frequency	Percentage
Yes	66	94.3%
No	4	5.7%
Total	70	100%

## Discussion

HU has been found to reduce the rate of VOC, ACS, RBC transfusion, and hospital stay duration, and has been shown to be associated with improved survival rates [15]. Our study demonstrated that the majority of pediatric patients were administered HU. A previous retrospective study from Riyadh showed that the administration of HU significantly reduced VOCs by more than half. Moreover, there was a significant decrease in the duration of hospitalizations, as well as a higher survival rate among children treated with HU [14]. Another similar study from Jeddah found that HU is the only therapeutic agent that alters the course of SCA patients [10].

In this study, we aimed to investigate the associated complications during painful crises among pediatric patients. This study showed most admissions were painful crises with ACS, followed by other complications such as splenic sequestration, stroke, and osteomyelitis. Our study findings are consistent with those of previous Saudi studies from Jazan, Al-Madinah Al-Munawarah, and Makkah Al-Mukarramah which revealed that the majority of complications were painful crises followed by ACS [8,11,16]. Our observations and results showed the associated complications of painful crises among pediatric patients with previous studies reporting similar complications.

The results showed that all patients experiencing one or two attacks were hospitalized for an average of four to nine days per year. This is similar to a study from Saudi Arabia. Regarding the duration of hospital stay and different factors, we found that the mean hospital stay was significantly higher among children who were admitted with infections and ACS and among children who were not administered HU [14]. Therefore, the duration of hospital stay depends on the severity of presenting complaints of SCA patient and their use of HU [14].

## Conclusions

This study highlights the frequency of painful crises and associated complications in pediatric SCA patients as well as HU use. We discovered that admission to the hospital was for an average of one to two times per year and the average hospitalization was four to nine days. The utilization of HU during painful crises was detected among the majority of SCA patients. We recommend further studies among SCA patients regarding the frequency of painful crises among different regions in Saudi Arabia.

## References

[1] Jastaniah W (2011). Epidemiology of sickle cell disease in Saudi Arabia. Ann Saudi Med.

[2] (2023). What is sickle cell disease?. https://www.nhlbi.nih.gov/health/sickle-cell-disease.

[3] Masom VR (1922). Sickle cell anemia, severe anemia with remarkable elongated shaped red blood cells. JAMA.

[4] Smith WR, Penberthy LT, Bovbjerg VE (2008). Daily assessment of pain in adults with sickle cell disease. Ann Intern Med.

[5] Creary M, Williamson D, Kulkarni R (2007). Sickle cell disease: current activities, public health implications, and future directions. J Womens Health (Larchmt).

[6] Makani J, Williams TN, Marsh K (2007). Sickle cell disease in Africa: burden and research priorities. Ann Trop Med Parasitol.

[7] Memish ZA, Owaidah TM, Saeedi MY (2011). Marked regional variations in the prevalence of sickle cell disease and β-thalassemia in Saudi Arabia: findings from the premarital screening and genetic counseling program. J Epidemiol Glob Health.

[8] Hawasawi ZM, Nabi G, Al Magamci MS, Awad KS (1998). Sickle cell disease in childhood in Madina. Ann Saudi Med.

[9] Piel FB, Steinberg MH, Rees DC (2017). Sickle cell disease. N Engl J Med.

[10] Alzahrani F, Fallatah AM, Al-Haddad FM, Khayyat ST, AlMehmadi WM, AlQahtani BG, Alamri RS (2021). Risk factors and complications among pediatric patients with sickle cell anemia: a single tertiary center retrospective study. Cureus.

[11] Alkot M, Almaghrabi WA, Al Najdi N, Al Otaib M, Shatla M, Abdelbaki H (2017). Prevalence of complications of sickle cell disease at Makkah Al-Mukaramah, Saudi Arabia. Ann Clin Lab Res.

[12] Abd Elmoneim AA, Al Hawsawi ZM, Mahmoud BZ, Bukhari AA, Almulla AA, Sonbol AM, Makhdoum AM (2019). Causes of hospitalization in sickle cell diseased children in western region of Saudi Arabia. A single center study. Saudi Med J.

[13] Abd El-Ghany SM, Tabbakh AT, Nur KI, Abdelrahman RY, Etarji SM, Almuzaini BY (2021). Analysis of causes of hospitalization among children with sickle cell disease in a group of private hospitals in Jeddah, Saudi Arabia. J Blood Med.

[14] Azmet FR, Al-Kasim F, Alashram WM, Siddique K (2020). The role of hydroxyurea in decreasing the occurrence of vasso-occulusive crisis in pediatric patients with sickle cell disease at King Saud Medical City in Riyadh, Saudi Arabia. Saudi Med J.

[15] Steinberg MH, McCarthy WF, Castro O (2010). The risks and benefits of long-term use of hydroxyurea in sickle cell anemia: a 17.5 year follow-up. Am J Hematol.

[16] Hazzazi AA, Ageeli MH, Alfaqih AM, Jaafari AA, Malhan HM, Bakkar MM (2020). Epidemiology and characteristics of sickle cell patients admitted to hospitals in Jazan region, Saudi Arabia. J Appl Hematol.

